# Association of *XPO1 *Overexpression with *NF-κB* and *Ki67* in Colorectal Cancer

**DOI:** 10.31557/APJCP.2019.20.12.3747

**Published:** 2019

**Authors:** Mohammed Aladhraei, Abdulla Kassem Al-Thobhani, Naravat Poungvarin, Prasit Suwannalert

**Affiliations:** 1 *Department of Pathobiology, Faculty of Science, *; 3 *Clinical Molecular Pathology Laboratory, Department of Clinical Pathology, Faculty of Medicine Siriraj Hospital, Mahidol University, Bangkok, Thailand, *; 2 *Department of Pathology, Faculty of Medicine and Health Sciences, University of Sana’a, Sana’a, Yemen. *

**Keywords:** Colorectal cancer (CRC), Exportin 1 (XPO1), NF-κB, Ki67, KPT-330

## Abstract

**Objectives:**

*Exportin 1(XPO1)*, a nuclear exporter protein, has been gaining recognition in cancer progression and treatment. This study aimed to evaluate the association between the overexpression of *XPO1 *with *NF-κB*, *Ki67* and clinicopathological characteristics in colorectal cancer (CRC) tissue samples and to explore the anti-proliferative effect of *KPT-330*, as *XPO1* inhibitor, in colorectal cancer cell line.

**Methods:**

Forty CRC tissue samples were analyzed by immunostaining for the expressions of *XPO1*, *NF-κB* and *Ki67* and then the anti-proliferative effect of the *KPT-330 *was also evaluated in HT29 colorectal cancer cell line.

**Results:**

*XPO1* overexpression was observed in 52.5% of CRC and significantly apparent with strong intensity in tumor cells compared to the normal adjacent epithelium (P<0.001). Regarding to the histopathological characteristics, the *XPO1* overexpression significantly associated with advanced tumor stages (P=0.049) and has great tendency towards moderate/poorly differentiated tumors. Although the *XPO1* overexpression was strongly associated with high* Ki67* expression (P=0.001), only *Ki67* expression showed significant association with tumor size (P=0.012). No significant association was detected between the *XPO1* overexpression and *NF-κB*, while the *NF-κB* positive expression was significantly associated with lymph node metastasis and *Ki67* expression at P=0.027 and P= 0.007, respectively. The in vitro experiments showed a great impact of *KPT-330*, as *XPO1* inhibitor, to inhibit cancer growth in dose and time dependent manner and significantly diminished the colony formation (P<0.001) of HT29 cells- associated with the expression of *Ki67* (P<0.001).

**Conclusion:**

*XPO1* overexpression and *NF-κB* expression may serve as potential biomarker associated with CRC pathogenesis and proliferation, while the *KPT-330* is effectively inhibited-colon cancer growth in vitro. Further studies considering the prognostication role of *XPO1 *overexpression in CRC are required.

## Introduction

Colorectal cancer (CRC) is one of the most common cancers, implicated as the third causative cancer morbidity and the fourth of cancer mortality worldwide (Torre et al., 2015). Despite recent advances in therapeutic approaches that have considerable practical value, the prognosis of more than 50% of CRC patients still remains poor (Bai et al., 2015). The uncontrolled expansion of proliferative activity propelled by the disruption of cellular homeostasis may play a major role to increase the growth and survival rates of CRC tumor cells (Sancho et al., 2004). *Exportin 1 (XPO1)*, also known as chromosome region maintenance 1 (CRM1) protein, is involved in the homeostatic of nucleocytoplasmic transport of over 200 known cargos, most of them are tumor suppressor and cell cycle regulatory proteins such as *p53, pRb, FOXOs, BRCA1/2* and inhibitor of *NF-κB* (Kau et al., 2004; Kashyap et al., 2016). In cancer, the disruption of *XPO1 *activity may be involved in the increasing of survival and proliferative rate activity of tumor cells (Turner and Sullivan, 2008). Preclinical data and many clinical trials used the developed new generation of selective inhibitor of nuclear export (SINE) compounds as *XPO1* inhibitor, such as *KPT-330*, has a property of low toxicity and good patients’ tolerability as it was seen in both hematological malignancies and solid tumors. This provided an excellent model in translational medicine for targeting of *XPO1 *activity in cancer therapy with promising efficacy in recent years (Abdul Razak et al., 2016). 

Association of *XPO1* overexpression with high tumor grade and advanced tumor stage (Noske et al., 2008; Shen et al., 2009) as well as with poor prognosis was noted in some tumor cancers such as gastric (Zhou et al., 2013), ovarian (Noske et al., 2008) and acute leukemia (Kojima et al., 2013), but little is known about such association between the *XPO1* overexpression and clinicopathological characteristics in CRC. In colon cancer cell lines, the *XPO1* increases the oncogenic activity of tumor cells through mislocalization of some proteins such as *p27* and survivin (Ferreiro-Neira et al., 2016; Heong et al., 2016). On the other hand, In CRC,* NF-κB* over-expression was considered as biomarker associated with worse 3 and 5 years overall survival (Wu et al., 2015). Therefore, exploration, in to what extent, the involvement of *XPO1 *overexpression in CRC characteristics in tissue samples, considering the histopathological characteristics and proliferation activity, in association with the *NF-κB* may be helpful in line of individualizing of patients treatment using *XPO1* inhibitor. Accordingly, in the present study, we tried to address the differences between the overexpression of *XPO1* in CRC tumor cells and adjacent normal epithelium. Then we explored the association between the *XPO1* overexpression and clinico-histopathological features as well as with *NF-κB *and proliferative marker, *Ki67*, in CRC tissue samples. Furthermore, we demonstrated the anti-proliferative effect of *XPO1* inhibitor, *KPT-330*, on HT29 colrectal cancer cell line.

## Materials and Methods

*Patients and clinicopathological data*

Samples in the form of formalin fixed paraffin embedded (FFPE) tissue blocks were collected randomly from 40 patients who underwent surgical resection and diagnosed as colorectal carcinoma between December 2016 and October 2017 at National Oncology Center (NOC), Sana’a, Republic of Yemen. Age and sex of the patients as well as tumor size were retrieved from the histopathological reports. The degree of histological differentiation of tumors were categorized according to percentage of the gland-like structures formation and grouped in to well and moderately/poorly differentiation (Xiao et al., 2013). The lymph node metastasis was also categorized in to negative (no regional lymph node metastasis) and positive (metastasis in to regional lymph nodes). The tumor stages were classified according to the TNM staging system, the American Joint Committee of Cancer (AJCC), and grouped in to stage I-II and stage III-IV. Hematoxylin and eosin (H&E) stained sections were reviewed to confirm the presence of >50% of tumor cells with adjacent normal epithelial for further study with immunohistochemistry (IHC) staining. Ethical approval for this study was obtained from National Health and Medical Research Committee (NHMRC), Republic of Yemen (B2/10-2017) and the informed consent was taken from all the CRC patients. 

*Antibodies and Reagents*

The anti-*XPO1* antibody was obtained from Santa Cruz Biotechnology, *XPO1/CRM1 (sc-74454)*, and the monoclonal anti-*NF-κB p65* antibody (phosphor S536) purchased from abcam, USA, while the anti-Ki67 antibody (MIB-1) was obtained from Cell Marque, USA. For in vitro experiments, colorectal cancer cell line HT29 was obtained from the American Type Culture Collection (ATCC). The HT29 cell was maintained in DMEM/F12 supplemented with 10% fetal bovine serum (FBS) (Sigma) and was cultured at 37°C in a humidified incubator of 5% CO_2_.* XPO1* inhibitor (KPT-330) was obtained from Karyopharm Therapeutics. DMSO was used as diluent control for all in vitro studies. 

*Immunohistochemical analysis*

Tissue sections from FFPE tissue blocks with 4μm thickness were cut and mounted on positive charge glass slides and processed by standard procedures for IHC in parallel with positive and negative controls for each antibody as described previously (Noske et al., 2008). Endogenous peroxidase activity was blocked using 3% hydrogen peroxide (H_2_O_2_) and antigen retrieval was done in citrate buffer (pH 6.0) using microwave oven. Then, the sections were incubated with monoclonal primary antibodies against *XPO1* (1:400), *NF-κB *(1:200) and *Ki-67* (1:500) overnight at 4°C, followed by a detection system HiDef Detection™ Amplifier and then a HRP Polymer Detector (anti-mouse/rabbit, Cell Marque, USA) for 10 minutes in each step. The DAB chromogen (3,3-diaminobenzidine tetrahydrochloride) then added with substrate for 5 minutes and the sections were washed in distilled water and then counterstained with hematoxylin for 2 minutes. The tissue sections were rinsed with PBS (pH 7.3) between each step during IHC processing. 

*IHC evaluation of XPO1, NF-κB and Ki67 expression*

Two independent investigators evaluated each section. The intensity scoring and positive staining cells were used to define the *XPO1* expression in CRC tumor cells and adjacent normal epithelium. The *XPO1 *expression was calculated by using the intensity score multiplied with positive cells score. The intensity of *XPO1* immunostaining scored as 0 (negative), 1 (weak), 2 (moderate), or 3 (strong) for either the nuclear, nuclear membranous and/or cytoplasmic area. The localization staining that diagnosed with positive cells scored according to the percentage of immunoreactive cells as 0 (none), 1 (<10%), 2 (10–50%) and 3 (>50%) and multiplied by the intensity score to give 0 score (negative expression), 1-3 (weak expression), 4-6 (moderate expression) and 7-9 (strong expression). The *XPO1* expression levels were categorized in to non-overexpression (negative, weak or moderate expressions) and over-expression (strong expression) (Gravina et al., 2015). 

The evaluation of *NF-κB (p65)* was done according the percentage of immunoreactive cells (quantity score) with the staining intensity in 10 high power visual fields of tumor cells. The percentage of immunoreactive tumor cells was evaluated as follow: no staining as 0, 1%-10% of cells stained as 1, 11%-50% cells stained as 2, 51%-80% cells stained as 3, and 81%-100% cells stained as 4. Staining intensity was rated on a scale of 0-3, with 0 = negative, 1 = weak, 2 = moderate, and 3= strong. The IHC staining of *NF-κB* was considered positive if the multiplied scored >3/12 (Long et al., 2008). The *Ki67* expression was assessed in 10 representative high power visual fields of tumor cells with cutoff value of <40% considered as low and ≥40% as high nuclear *Ki67* expression (Salminen et al., 2005).

*Cell growth inhibition assay*

Cells were seeded at density 10^4^ cells/well in 96-well plates, then the cells were treated next day with series doses below and above their IC_50_ concentrations of KPT-330, 0.25, 0.5, 1, and 2μm/L and then further incubated for 24, 48 and 72 hours respectively. Thereafter, the cells were subjected to cell proliferation analysis in consecutive days using MTT [3-(4,5-dimethylthiazol-2-yl)-2,5-diphenyltetrazolium bromide] assay and incubated for 4 hours. After that, the cells were lysed by 200 µL of DMSO. Then the spectrophotometric absorbance of the samples was determined using a microplate reader (Bio-Rad) (Ferreiro-Neira et al., 2016). 

*Colony formation assay*

The ability of *KPT-330* to inhibit the HT29 cell lines colonies formation was assessed by the colony formation assay as described previously (Niu et al., 2015). Briefly, 10^2^ HT29 cells were seeded into 24-well plates in triplicates and then treated with vehicle control (DMSO) and series doses of *KPT-330*, 0.5, 1, 2µmol/L) for 24 hours. The culture medium was changed and the cells were incubated to allow colonies formation. After two weeks, cells were fixed with 5% glutaraldehyde in PBS and stained with 0.1% crystal violet. The numbers of colonies formed were confirmed by manual counting for each dose.

*Immunocytochemical (ICC) expressions of Ki67 in HT29-KPT-330-treated cells*

The HT29 cells seeded on cover slips in 6-well at density of 10^5^ cells, incubated overnight and then subjected to increasing doses of *KPT-330* and incubated for further 48 hours. The cells then washed with PBS, fixed in cold acetone immersed in hydrogen peroxide for 10 minutes. After that, the cells incubated with anti-Ki67 (1:1,000) antibody for one hour followed by incubation in the HiDef Detection™ Amplifier and HiDef Detection™ HRP Polymer Detector for 10 minutes in each. The cells then incubated with DAB-chromogen system for 3-5 minutes followed by counterstain in hematoxylin. Scoring of Ki67 was done depending on the percentage of positive cells (0%­ - 100%) of the total cells numbers in high-power fields, as in previous study (Zu et al., 2012).

*Statistical analysis*

In this study, all analysis was performed using SPSS version 18 statistical software program (SPSS Inc., Chicago, IL, USA). Chi-square and Fisher’s exact tests were used to find the association between the overexpression of *XPO1, NF-κB* and *Ki67* with clinico-histopathological factures. Mann-Whitney U test was used with continuous variables. All cell culture experiments were performed in triplicate and repeated at least three times while the one-way ANOVA was used to compare the mean between groups. The P values <0.05 was considered statistically significant difference. 

## Results

*Expressions of XPO1, NF-κB and Ki67 in CRC*

**Figure 1:**
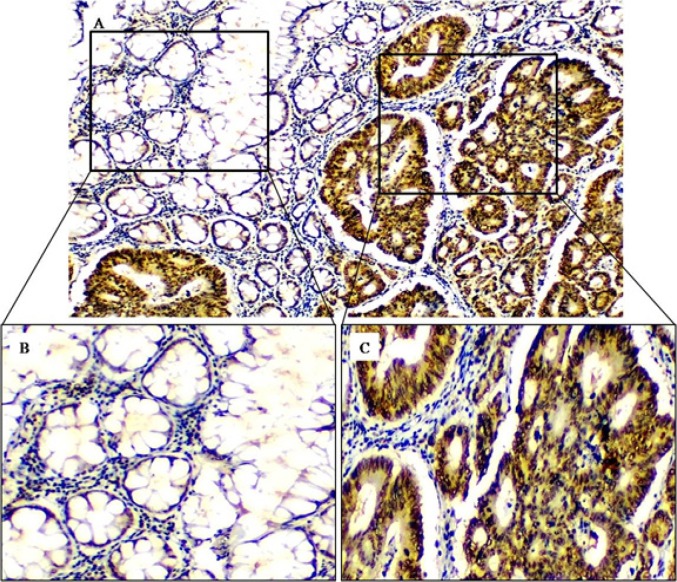
XPO1 Expression in Adjacent Normal Epithelium (A1 and B) and Tumor Cells (A2 and C). (Original Magnification- 200x and 400x, respectively).

The average age of the patients was 49.9 years (range 24-80), with 55% (22/40) were females. After we used the IHC analysis, the majority of *XPO1* expressions were nuclear and/or nuclear membrane with weak to moderate diffuse cytoplasmic staining. The *XPO1* expression was identified and scored as negative (15%), weak (12.5%), moderate (20%) and strong (52.5%). The only strong score was considered as *XPO1* overexpression (52.5%) while the 47.5% were considered as *XPO1* non-overexpression. Although there was no big differences in the frequency of the* XPO1* immunostaining between the tumor cells and normal adjacent epithelium, the intensity of *XPO1* expression was abundant, intense and more apparent in tumor cells compared to the adjacent normal epithelial with significant difference (P<0.001) (Table 1 and Figure 1). The positive *NF-κB* expression was noted in 32.5% (13/40) while the Ki67 was observed as high expression in 72.5% (29/40) of CRC tumors (Table 2). 

*Association between the XPO1 overexpression and NF-κB, Ki67 with clinicopathological features of CRC tissue samples*

**Figure 2:**
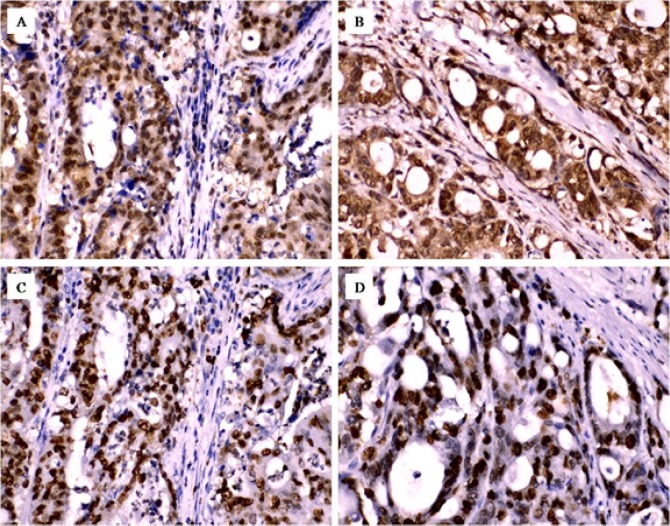
Association of XPO1 Overexpression and *NF-κB* with *Ki67*. The IHC expressions of both *XPO1* (A) and NF-κB (B) show highly concordance of immunoreactivity within the tissue sections expressed* Ki67* (C and D respectively) (original magnification- 400x).

**Figure 3:**
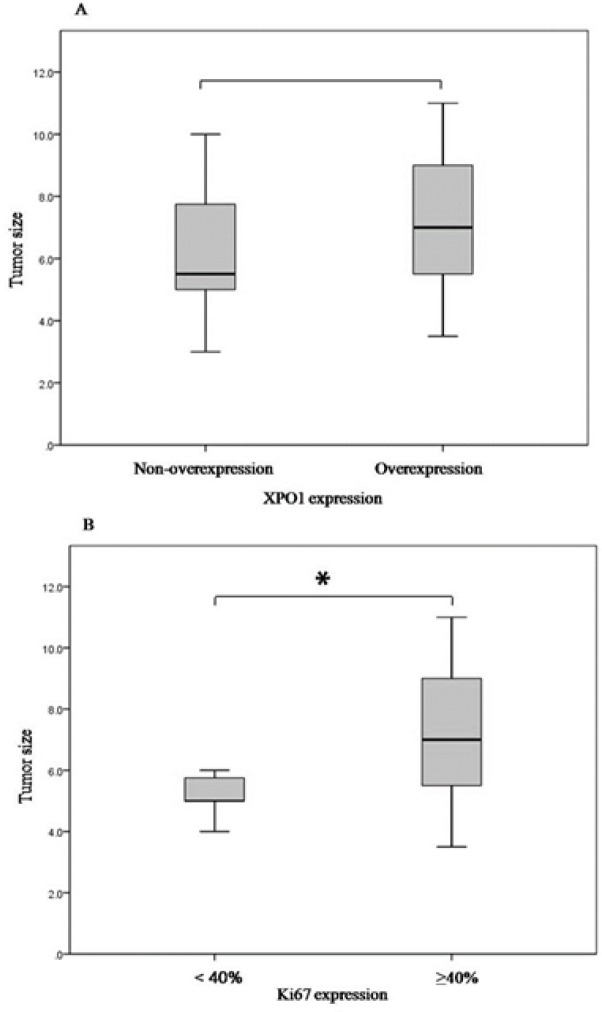
Correlation between the *XPO1* overexpression and *Ki67* with tumor size. Mann-Whitney test demonstrate that the tumors with larger size exhibit significant high *Ki67* expression (A) (P=0.012) and to some extent the *XPO1* overexpression (B) (P=0.168). * represents the significant statistical differences between groups at P<0.05

In this study we identified a high tendency of *XPO1 *overexpression in moderately/poorly differentiated tumors and was significantly associated with advanced tumor stage (III/IV) (P=0.059 and P=0.049, respectively) (Table 2). Although the *XPO1* overexpression was frequently noted in tumors with increasing numbers of positive lymph nodes metastasis, this tendency did not reach to the statistical significance difference (P=0.308). Significantly, we found a strong concordance between the *XPO1 *overexpression and high *Ki67* expression in the tumor cells within the most tissue sections that showed immunoreactivity for both *XPO1* and *Ki67*, and the *XPO1* overexpression was strongly associated with the high *Ki67 *expression (P=0.001) (Table 2 and Figure 2). On the same manner, the CRC tumors sizes in this study ranges from 3 to 11 cm with mean 6.6 cm and we noted that the high nuclear Ki67 expression was associated with an increase tumor size (P=0.012) but not *XPO1* (P=0.168), with Mann-Whitney test, as it is illustrated in Figure 3. In this study, although the *XPO1* overexpression was noticed in 8/13 of positive NF-κB expression tumors, the statistical significant association between the *XPO1* overexpression and *NF-κB* was not found (P=0.427) (Table 3). However, most of the *NF-κB* positive tumors (11/13) were involved with positive lymph nodes metastasis with significant difference (P=0.027) and showed strong association with the high *Ki67* expression (P=0.007) (Table 2 and Figure 2)

*KPT-330 inhibits cell proliferation, colony formation and Ki67 expression of HT29 cells*

**Figure 4:**
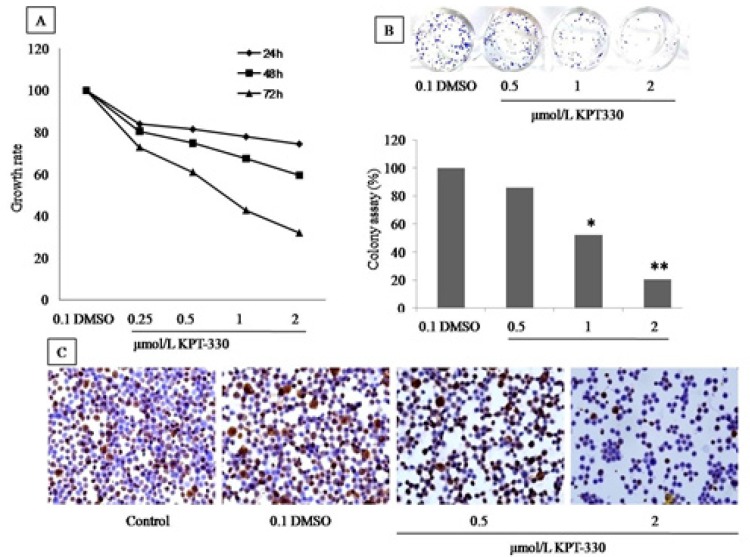
*KPT-330* Inhibits the Growth and Proliferation of HT29 Cell Line. MTT assay (A), note the decreases of rate cell growth after 24, 48 and 72 hours of incubation while the colony formation assay (B) evince the ability of* KPT-300 *to inhibit the colonies formation. The ICC (C) staining shows reversible results of cell-expressed-ki67 and *KPT-330 *at doses 0.5 to 2µmol/L. *,** represent the statistical differences between the treated and untreated cells (P<0.001 and P<0.0001, respectively)

As shown at Figure 4A, the increased of KPT-330 serial concentrations from 0.25, 0.5 and 1 to 2 µmol/L induced the cells growth inhibition after 72 hours of incubation. The IC_50_ value was 0.9 µmol/L in HT29 cells. These results revealed that, when the* KPT-330* dose exceeds 1 µmol/L and the action of time was >24 hours, the growth of cells was inhibited in a time and dose-dependent manner. Further evaluation of the long-term effect of* KPT-330* on HT29 growth, the clonogenic assay was performed for 2 weeks. The results revealed that the colonies formation decreased significantly by 86%, 52% and 21% in response to *KPT-330* doses of 0.5, 1 and 2 μmol/L, respectively (Figure 4B). Moreover, the *Ki67 ICC *staining of *KPT-330*-treated-HT29 cells showed that the Ki67 positive cells in the *KPT-330* treated groups was significantly reduce compared to the untreated (Figure 4C). These results were consistent with the MTT and colony formation assays results, confirming that the *KPT-330* inhibits the proliferation of HT29 colon cancer cell line in dose and time dependent manner.

**Table 1:** Expression of *XPO1* in CRC Tumors Cells and Adjacent Normal Epithelium

XPO1	Number of patients (%)		P value
expression	Normal (%)	Cancer (%)	
Negative	3 (7.5)	6 (15.0)	
Weak	18 (45.0)	5 (12.5)	
Moderate	15 (37.5)	8 (20.0)	
Strong	4 (10.0)	21 (52.5)	<0.001^*^

**Table 2:** Association of *XPO1* Overexpression and *NF-κB *with Clinicopathological Features and *Ki67* Expression

Clinicopathological	Patients	XPO1 expression		P-value	NF-κB expression		P-value
features and Ki67 expression	No (%)	Nonoverex-pressio	Overexpr-ession		-ve	+ve	
Tumor differentiation				0.059			0.906
Well	19 (47.5)	12	7		13	6	
Moderate/Poor	21 (52.5)	7	14		14	7	
Lymph node metastasis				0.366			0.027*
Negative	16 (40)	9	7		14	2	
Positive	24 (60)	10	14		13	11	
Tumor stage				0.049*			0.286
I–II	11 (27.5)	8	3		4	7	
III-IV	29 (72.5)	11	18		7	22	
Ki67				0.001**			0.007**
<40%	11 (27.5)	10	1		11	0	
≥40%	29 (72.5)	9	20		16	13	

*,** represent the statistical P<0.001 and P<0.0001, respectively.

**Table 3:** Association between the *XPO1* Overexpression and* NF-κB* in CRC

NF-κB expression	Patients No (%)	*XPO1* expression		P value
		Nonoverex-pressio	Overexpr-ession	
Negative	27 (67.5)	14	13	
Positive	13 (32.5)	5	8	0.427

## Discussion

*XPO1 *is the major mammalian exporter protein that facilitates the nucleocytoplasmic transport of over 200 tumor suppressors and cell cycle regulatory proteins. Its overexpression reported in several types of tumors and was correlated with aggressive behavior and poor survival (Sun et al., 2016). Furthermore, in recent years the *XPO1* gains a great attention after launching a next-generation selinexor (*KPT-330*) as selective inhibition of nuclear export of *XPO1* with good properties regarding low toxicity in vivo and good tolerability by the patients (Lapalombella et al., 2012; Hing et al., 2016). In CRC, little is known about the overexpression of *XPO1* in tissue samples and its association with histopathological features, *NF-κB *and *Ki67*. Our previous study concerned of *p53*, reported that the *XPO1* positivity was associated with loss of *p53* expression in CRC tumors with lymph node metastasis (Aladhraei et al., 2019). In the current study, we noticed a significant apparent of *XPO1* overexpression in CRC tumor cells compared to the adjacent normal epithelium as it was reported in many other types of cancers such as esophageal (van der Watt et al., 2014), gastric (Subhash et al., 2018), lung (Gao et al., 2015), and ovarian (Noske et al., 2008) cancers, as well as leukemic cells (Kojima et al., 2013). The overexpression of *XPO1* within the tumor cells may reflect its abundance and suggests a gain-of-function or oncogenic activity in line of further CRC pathogenesis (Conforti et al., 2015). Crochiere et al., (2016) suggested that the abundance of *XPO1* within the tumor cells might be able to predict the drug resistance.

As it is known, the tumor grade is considered as stage-independent prognostic factor in CRC, and the poorly differentiated tumors are associated with poor patient survival (Fleming, et al., 2012). In this study we identified a high tendency of *XPO1* overexpression towards the moderate/poorly differentiated tumors as well as increase of *XPO1* overexpression frequency in advance tumor stages III-IV with significant difference. Although Shintani et al., (2016) studied the *XPO1* expression in CRC tissue samples, they did not find such association in CRC tissues which may attribute to the large differences in the sample sizes in each of well and poorly differentiated tumor groups; others were found such association in other types of cancers such as ovarian cancer (Noske et al., 2008), glioma (Shen et al., 2009) and the *XPO1* expression was increased from well to poorly differentiated breast tumors (Yue et al., 2018). Additionally, we noted that the poorly differentiated tumors were significantly increased numbers of metastatic lymph nodes, which goes in line with a cohort study results of 124,180 CRC patients by Ricciardi et al., (2006) who concluded that the poorly differentiated tumors were much more likely to be with lymph node positive than well-differentiated tumors. In this study, most of moderately/ poorly differentiated tumors with *XPO1* overexpression were involved by lymph node metastasis. Moreover, the clinical trial carried by Mau-Soerensen et al., (2014) among advanced metastatic CRC patients and used oral* KPT-330* for 28 days confirmed the valuable of *XPO1* inhibitors in line of disease stability for advanced staged CRC patients. Collectively, these results may add insights of *XPO1* involvement in the CRC pathogenesis and progression leading to poorly differentiated tumors and advance tumor stages. In some tumors such as lung cancer and mantle cell lymphoma cells, the *XPO1* inhibitors modulate the *NF-κB* activity through trapping of *IκB* in the nucleus, which is target of *XPO1* for nucleocytoplasmic transportation, leading to repression of *NF-κB* activity over time which contributes in the growth suppression and apoptosis induction (Zhang et al., 2013). In this study we found that the* XPO1* overexpression was associated, to some extent, with the *NF-κB* positive expression without significant difference. On the other hand, it has been proved that the activated *NF-κB* contributes in the progression of CRC through upregulation expression of diverse target genes that are involved in inflammation (cytokines), cell proliferation (*Cyclin D1*), angiogenesis (*VEGF, IL-8, COX2*), and metastasis (MMP9) (Wang et al., 2009; Xie et al., 2019) make it an interesting tumor marker in CRC pathogenesis. In this regard, the results of our study showed that the *NF-κB* expression was significantly associated with positive lymph node metastasis. Furthermore, strong significant association between the expressions of NF-κB and high *Ki67* expression was identified. These findings were consistent with in vitro studies (Lu et al., 2016) as well as with *NF-κB *activity role in CRC tumors (Meteoglu et al., 2015). These results add an insight in line of *NF-κB *involvement in CRC metastasis and proliferation makes a rational for targeting of *NF-κB* in CRC as it was reported (Sakamoto and Maeda, 2010). It is well known that the sustained proliferation is one of the cancer hallmarks acquired during cancer development and progression (Hanahan and Weinberg, 2011). In this regard, we identified that the overexpression of *XPO1* was strongly associated with *Ki67* expression, which in turn reflected the implication of *XPO1* overexpression in mislocalization of essential cell cycle inhibitory proteins such as *p27*, *p53*, cyclins and some apoptotic proteins (Nguyen et al., 2012; Niu et al., 2015) lead to unregulated cell division and increased tumor size. Despite ascending tendency of *XPO1* expression was noticed with increased tumor size, our study did not find a direct association between the *XPO1* overexpression and tumor size; while the high *Ki67* expression was significantly noticed in larger tumor sizes. The strong association between the overexpression of *XPO1* and high *Ki67* expression in CRC patients’ tumors was in concordance with our in vitro results that confirmed the anti-proliferative effect of *KPT-330*. The *KPT-330* induced the growth inhibition and suppressed the HT29 colorectal cancer cell line proliferation in dose and time dependent manner as shown by the MTT assay. Furthermore, the experiments showed a great stability of *KPT-330* in vitro, which means a continuous inhibition of growth over the time during incubation and persisted for up to 72 hours. This explains the powerful binding of *KPT-330* in slowly reversible action with *XPO1* leading to inhibition the binding between the cargoes proteins and *XPO1* in HT29 cancer cell line which in turn induce nuclear retention of cell cycle regulatory proteins, inhibits the proliferation and may initiate the apoptosis (Draetta et al., 2011; Senapedis et al., 2014). The long-term effect of *KPT-330* on the HT29 colony formation was significantly seen as a decrease of colonies formation with increase of KPT-330 concentration. These findings were further supported by the evaluation of *Ki67* expression by ICC in *HT29-KPT-330*-treated cells. The number of HT29 cells with Ki67 expression was dramatically and significantly decreased in *HT29 KPT-330*-treated groups compared to the control, and this confirmed the anti-proliferative effect of *KPT-330*. In fact, the inhibition of *XPO1* activity induced G1 cell cycle arrest with the loss of S, G2, and M phases within hours of application followed by increase of nuclear cell cycle inhibitory proteins such as *p27* and p53 (Niu et al., 2015). Normally, the *Ki67* is degraded and decreased continuously in G0 and G1, which indicate the decrease of protein synthesis and then accumulate from S to M phases, which indicate an increase of cells proliferative activity. The increase of the sub-G1 fraction within the cells may an indicative of apoptosis that explains the *KPT-330* action in time dependent manner (Sobecki et al., 2017; Miller et al., 2018). This result explains the reduction of HT29-cells-expressed-*Ki67* with increase doses from 0.5 µmol/L to 2 µmol/L of *KPT-330* after 48 hours of incubation. The above results fall in-line with the published data about the *KPT-330* ability to sequester the cargoes such as tumor suppressor proteins within the nucleus and leads to cell cycle arrest and proliferation inhibition. The anti-proliferative effect of* XPO1* inhibitors were reported in different types of cancer cell lines such as pancreatic cells (Azmi et al., 2017), liver cells (Zheng et al., 2014), prostate cells (Gravina et al., 2015), and gastric cells (Subhash et al., 2018) as well as in colon cancer cell lines (Draetta et al., 2011; Niu et al., 2015). In conclusion, an apparent of *XPO1* overexpression in CRC tumor cells compared to the adjacent normal epithelium as well as the association of *XPO1* overexpression with advance tumor stages, tumor differentiation and high *Ki67 *expression may reflect its potential involvement in CRC pathogenesis which can be inhibited by *KTP-330*. The *XPO1* overexpression and *NF-κB* expression may serve as potential biomarker associated with CRC proliferation and pathogenesis, therefore, further studies regarding the *XPO1* overexpression prognostication in CRC patients may be recommended. 
